# Overcoming barriers to NHS adoption of innovative IPC products: A qualitative study of SMEs in the Liverpool city region

**DOI:** 10.1371/journal.pone.0331688

**Published:** 2025-09-16

**Authors:** Rocío Villacorta Linaza, Janet Hemingway, Adam P. Roberts, Becky Jones-Philips, Miriam Taegtmeyer, Richard L. Wright, Daire Cantillon, Maria Moore, Russell Dacombe, Ezekiel Boro, Aaron Argomandkhah, Carolina Velasco, Nicholas Feasey

**Affiliations:** 1 Department of Tropical Disease Biology, Liverpool School of Tropical Medicine, Liverpool, United Kingdom; 2 Department of Education, Liverpool School of Tropical Medicine, Liverpool, United Kingdom; 3 Department of Vector Biology, Liverpool School of Tropical Medicine, Liverpool, United Kingdom; 4 Enterprise and Innovation Unit, Liverpool School of Tropical Medicine, Liverpool, United Kingdom; 5 Department of Clinical Sciences, Liverpool School of Tropical Medicine, Liverpool, United Kingdom; 6 Behaviour 2 Business Ltd, Edinburgh, United Kingdom; 7 Department of International Public Health, Liverpool School of Tropical Medicine, Liverpool, United Kingdom; 8 Malawi-Liverpool-Wellcome Programme, Kamuzu University of Health Sciences, Blantyre, Malawi; 9 School of Medicine, University of St Andrews, St Andrews, United Kingdom; Ataturk University, TÜRKIYE

## Abstract

Healthcare-associated infections (HAIs) result in prolonged hospital stays and an increased incidence of infections caused by antimicrobial-resistant bacteria. Small and Medium Enterprises (SMEs) play a crucial role in developing innovative Infection Prevention and Control (IPC) solutions, but they face substantial challenges in navigating the complex NHS procurement system. This study, carried out in the Liverpool City Region in 2022, investigated these barriers. The methodology involved qualitative data collection through an online survey and five semi-structured interviews with SMEs involved in IPC innovation. The survey targeted 114 SMEs, and the interviews were conducted remotely with management teams. Data were analysed thematically using NVivo software, allowing for the identification of key barriers and recurring themes across the dataset. Key challenges identified included high market-entry costs, navigating complex regulatory and procurement frameworks, and limited access to key NHS stakeholders. These issues were compounded by fragmented decision-making processes within NHS Trusts, making it difficult for SMEs to secure product adoption. Despite these barriers, SMEs remain committed to innovating IPC solutions, driven by the potential to improve patient care and address antimicrobial resistance. This report recommends streamlining support mechanisms for SMEs, improving access to NHS decision-makers, and advocating for policy reforms to simplify the procurement process. By facilitating collaboration between SMEs, the NHS, and other stakeholders, the adoption of innovative IPC products can be accelerated, ultimately benefiting patients and addressing the significant public health threats posed by HAIs and antimicrobial resistance.

## Introduction

Healthcare-associated infections (HAIs) are acquired while receiving care and treatment in health facilities. Affected patients tend to have longer hospital stays and higher rates of infection with antimicrobial resistant (AMR) bacteria [1,2]. In 2016/2017 (as per most recent published point prevalence survey – due to be updated 2024), there were an estimated 653 000 HAIs in hospitals in England, of which 23,000 patients died as a result of their infection. These infections were estimated to account for a total of 5.6 million occupied hospital bed days and 62,500 days of absenteeism among front-line Health Care Professionals (HCPs). The cost of managing a patient who acquires a healthcare-associated infection (HCAI) is approximately three times higher than managing a patient without one. Furthermore, in 2016/2017, HAIs were estimated to have cost the NHS £2.1 billion HAI control is achieved by a complex combination of policies, infrastructure, organisation, and knowledge [3]. Infection Prevention and Control (IPC) teams are responsible for making healthcare settings safe for staff and patients through the strategic deployment of efficacious hygiene products and interventions to disrupt and/or trace transmission events. There is a need to more rapidly develop, evaluate and, where appropriate, deploy IPC technologies due to the public health risk posed by the constantly evolving nature of HAIs and associated antimicrobial resistance (AMR).

A combination of multiple integrated evidence-based interventions is essential to reducing the burden of HAI and AMR [4]. These include antimicrobial stewardship activities, along with rapid, high-quality diagnostics, appropriate and timely antimicrobial treatment, and prevention of infection through transmission control strategies. The 2015 Global Action Plan on Antimicrobial Resistance recommends a multi-pronged approach to IPC and has been adopted and adapted by 117 countries, including the UK, where investing in innovation, supply and access are one of three key pillars of AMR reduction [5–9].

NHS England states: “The NHS is a complex system, which can sometimes make it difficult to understand – especially working out who is responsible for what. It’s made up of a wide range of different organisations with different roles, responsibilities, and specialities”. As an example, in LCR alone, there are 86 GP practices collaborating across 12 neighbourhoods and 11 Primary Care Networks, linked to seven NHS provider trusts, one adult acute care trust, a children’s acute trust, a women’s acute trust, and three specialist trusts. The difficulty of managing this complexity, particularly cost and human resources, has come under even more pressure due to COVID-19, privatisation, and other recent health reforms [10–16].

One area with significant innovation potential is the infection prevention and control arena. Effective IPC practices are critical for reducing adverse public health outcomes and reducing the economic burden on healthcare systems such as the NHS and innovations in this space might take the form of novel chemical or biological hygiene products, novel materials and novel IT and data analysis approaches. By leveraging cutting-edge technologies, transformative approaches, and dynamic market access and use approaches, innovation can support IPC strategies, reducing the prevalence and transmission of AMR pathogens and thereby reducing the risk of HAIs and their associated economic impact [17]. Health and Life Sciences (HLS) innovation is particularly relevant to healthcare systems such as the NHS, which must balance patient outcomes against a precarious public funding model, requiring demonstrable and significant health economic benefits for the implementation of new interventions [18].

The UK innovation pathway involves diverse stakeholders such as commercial sector, research institutions, financial institutions, charities, government, and the Medicines and Healthcare products Regulatory Agency (MHRA), before a product is ready for adoption by the NHS. Procurement by the NHS is complex and multilayered, and in some instances, prohibitive for new entrants [10]. However, recent experiences in the COVID-19 pandemic demonstrated that the collaborative processes required for rapid innovation and deployment within the NHS are achievable despite these barriers, the best example being the Oxford–AstraZeneca SARS-CoV-2 vaccine [19–26].

However, in reality, many innovative HLS interventions, especially those supporting IPC strategies, encounter significant barriers along the innovation pathway. This is true for products developed by SMEs and for products developed by larger biotech. Despite £15.5 billion of central government spend on SMEs in 2019/20, almost half (47%) of UK SMEs in health-related sectors considered market-entry cost as one of the barriers when it comes to pursuing innovative activity especially in the current economic setting and the slower recent growth of NHS funding [27–32].

Such are the barriers to market entry, a reported 24% of UK-based HealthTech SMEs aim to launch their innovations in the US, rather than the UK, due to its market size and favorable regulatory environment [33]. Recognition of the challenges faced by SMEs, such as budget silos that make certain parts of the system reluctant to invest in innovations that generate savings elsewhere, has prompted action. Innovations are often assessed based on minimal cost, and for suppliers, pathways to market remain unclear. Furthermore, limited access to rapid funding for adoption, the complexity of decision-making processes within the NHS, and its organisational fragmentation are significant factors limiting the adoption of new solutions. [34–37]. To address these challenges, a range of local, regional, and national initiatives (Table 1) have been expedited, specifically designed to provide financial and technical incentives for navigating the innovation pathway [38].

**Table 1:** Local, regional and national NHS innovation pathways [12].

Scale	Example
Local	• NHS vanguard – NHS England » Vanguards
	• Academic Health Sciences Centres – https://www.england.nhs.uk/aac/what-we-do/innovation-for-healthcare-inequalities-programme/academic-health-science-centres/
Regional	• Infection Innovation Consortium (iiCON) – Home | iiCON (infectioninnovation.com)
	• Health Innovation Networks – Home – The Health Innovation Network
	• Applied Research Collaborations – • Applied Research Collaborations – Research themes, national leadership areas and national priority areas | NIHR
	• Regional Medicines Optimisation Committees – NHS England » Regional Medicines Optimisation Committees Advice
National	• Accelerated Access Collaborative – NHS Accelerated Access Collaborative (england.nhs.uk)
	• NHS Digital – Home – NHS England Digital
	• NHSX – NHSX: new joint organisation for digital, data and technology – GOV.UK
	• NIHR Funding Schemes – Funding opportunities | NIHR
	• NIHR Artificial Intelligence Scheme – Artificial intelligence funding | NIHR
	• NHS Innovation Accelerator – Home – NHS Innovation Accelerator (nhsaccelerator.com)
	• Innovate UK – Innovate UK – UKRI
	• Medilink UK – Home – Medilink UK
	• NHS Clinical Entrepreneur Programme – NHS Clinical Entrepreneur Programme – Transforming healthcare through innovation (nhscep.com)
All levels	• Angel Investors – UK Angel Investment Network – Business Angels, Entrepreneurs & Angel Investors
	• Venture Capital
	• Start-up Accelerator
	• Equity Crowdfunding Incubator

To address this gap, the Liverpool School of Tropical Medicine (LSTM) set out to establish the Infection Innovation Consortium (iiCON). iiCON’s purpose is to accelerate products to market and bridge the gap in the infection innovation ecosystem between industry, academia, and the NHS. Based in the Liverpool City Region (LCR), iiCON is strategically positioned in one of the UK’s leading hubs for infection control research. With its longstanding expertise in infectious disease, LSTM and iiCON are uniquely equipped to drive innovation in Infection Prevention and Control (IPC) technologies and combat antimicrobial resistance [39]. The consortium, a network of academic, NHS, and SMEs, and large enterprises from the Northwest, leverages its regional expertise to support the discovery and development of new IPC products. In March 2022, iiCON organised a workshop convening multiple stakeholders from industry, academia, and the NHS in Liverpool, reflecting its commitment to improving healthcare product access to the NHS. This paper addresses the resultant stakeholder request for a more detailed analysis of the barriers and obstacles SMEs face in navigating the NHS procurement process.

## Materials and methods

**Fig 1:**
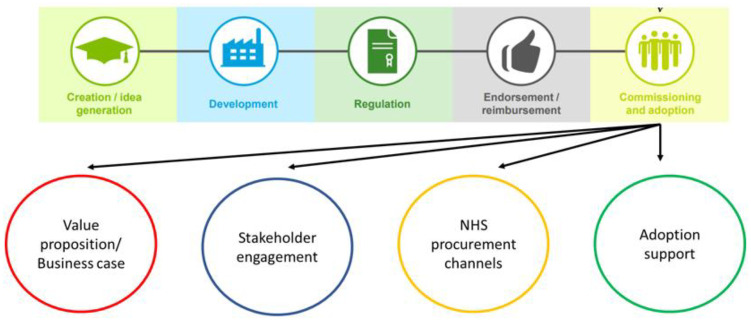
Key items to take into consideration to complete the “adoption” stage [39].

The research employed a qualitative methodology, gathering data through both semi-structured interviews and an anonymous survey to capture the perceptions and experiences of SMEs. The *Office for Life Sciences guide* to navigating the innovation pathway in England (2016) was used as an analytical framework to inform the development of a questionnaire specifically designed for SMEs. This guide provides a comprehensive overview of the innovation pathway for products to be used by the NHS in England. This includes the key stages of idea generation, development, regulation, reimbursement, endorsement (such as health technology assessments), commissioning, and adoption (**Fig 1).** The guide also outlines key organisations and contacts relevant to each stage and provides a checklist of critical considerations for stakeholders navigating the innovation process. This structured approach supports the progression of innovations from concept to adoption, helping to streamline the often-complex NHS procurement and adoption pathways [40]. These five domains, along with additional questions from surveys designed for SMEs [41,42], guided the design of an anonymised online survey with both closed and open questions and semi-structured interview questions (survey questions and topic guide available in Supplemental Materials).

In June 2022, an anonymised online survey was designed using Microsoft Forms [43] to collect both qualitative and quantitative data. The survey targeted SMEs involved in healthcare innovation within the Northwest of England. The survey was distributed on 13th July 2022–114 SMEs: 84 recipients were direct contacts within the iiCON database, while 30 were contacted via generic email addresses. The survey link was also shared with the local business community through the Liverpool Growth Platform’s July 2022 Health & Life Sciences newsletter and the Northwest Innovation Agency’s August newsletter [44,45].

Simultaneously, five SMEs were purposively chosen for semi-structured interviews based on three criteria: the NHS adoption of their innovative products, company size, and receipt of funding for part of their innovation journey. The interviews took place between 11th and 28th July 2022. Prior to the interviews, the primary researcher (RVL) provided detailed information regarding the research and obtained signed consent from participants. These interviews were conducted remotely via Microsoft Teams, lasting up to one hour each. While some of the SMEs had previous connections with iiCON, the primary researcher was not personally familiar with them. To avoid bias, standardised interview guides with open-ended questions were used (**Table 2),** and reflexivity was maintained throughout the process. All interviews were recorded and later transcribed using Microsoft Teams’ transcription functionality. Although only five SMEs were interviewed, the sample was selected purposively to ensure diversity in company characteristics and relevance to the research aims. In qualitative research, smaller sample sizes are acceptable when participants are information-rich and data collection reaches thematic saturation [46]. This approach supports depth over breadth and aligns with established qualitative methodology standards.

**Table 2:** Semi-structured guiding questions.

Semi-structured guiding questions
1. Could you please describe the work your company does?
2. Could you please explain the barriers you have/are facing while trying to adopt your product to the NHS?
3. Could you please explain your experience working with different trusts and if the barriers/challenges have been the same for each trust?

Data from the survey and interviews were analysed using an inductive approach, with collection and analysis occurring simultaneously. Initial insights from the survey informed the interviews, allowing an iterative refinement of the data collection process. The primary researcher analysed the qualitative data using NVivo 12 software. Text was coded into 18 analytical categories, which were grouped into five key themes. These themes were derived through constant comparison and iterative coding, with attention paid to the recurrence of patterns across different participants. Thematic saturation was assessed during analysis and considered achieved when no new themes or insights emerged from the final interviews [47,48]. This thematic analysis helped in identifying patterns across both data sources. Continuous discussion with the wider iiCON working group ensured that emerging findings were challenged, gaps were addressed, and final themes were agreed upon. No significant discrepancies were observed between the survey and interview responses, further validating the results.

This comprehensive analysis allowed the triangulation of findings between the two methods, although participant anonymity in the survey limited the ability to disaggregate the data by participant type.

### Ethics statement

Ethical approval was granted by the Liverpool School of Tropical Medicine Research Ethics Committee (Research Protocol 22–044). Informed consent was obtained from all participants. For the semi-structured interviews, a written consent form was sent to the participants a week in advance, and signed before the interview took place. For the online survey, informed consent was implied by participants’ completion of the survey after being provided with detailed information on the purpose of the survey, confidentiality measures, and the use of the data at the beginning of the survey.

## Results

In total, 12 SMEs answered the online survey, 10 of which were based on LCR. All SMEs were remotely interviewed using Microsoft Teams and were based in LCR. 85% of the innovative products produced by participating SMEs were for IPC.

Adoption of innovative products into the market, including the crucial NHS market, poses multiple challenges for SMEs. The analysis revealed the following analytical categories within five key themes: cost, procurement and supply chain frameworks, access to NHS stakeholders, and decision-making processes. To preserve the anonymity of participants, we have omitted specific details about the size of the company, the nature of their products, and the funding they received.

**Fig 2:**
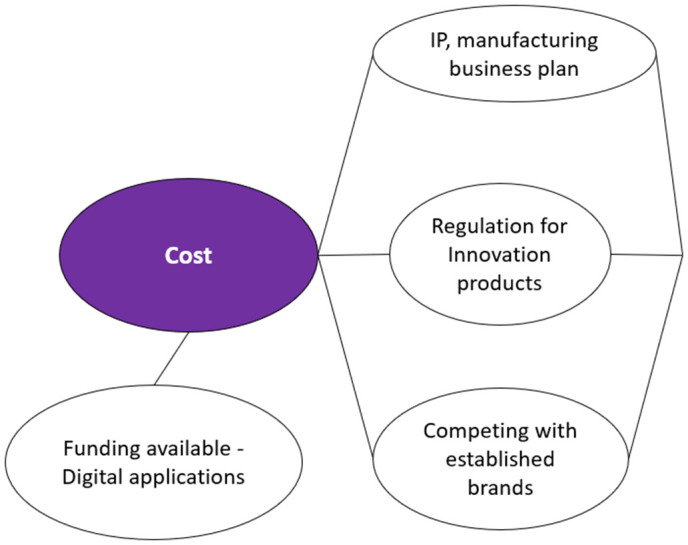
Visual representation of the “Cost” analytical categories.

Market Entry Costs as Key Barrier to Health Innovation One of the primary hurdles to the ultimate uptake of innovative health products for IPC was described by participants as being the product development cost as represented (**Fig 2**).

The costs associated with establishing intellectual property (IP) and trademark place a substantial burden on SMEs.

*“Besides the sale of the product, the intellectual property, whether it’s a patent or a trademark, is the most valuable asset that you have”.* (semi-structured interviewee 2)

Other costs mentioned by participants related to regulation, accreditation, testing, verification, manufacturing, development, and business planning.

*“If you believe in it and you may wish to make it your vocation to commercialise. That can be quite daunting, and I don’t think it’s readily understood how much money it costs...”* (Semi-structured interviewee 2)

The diversity of regulations for different product categories was highlighted as causing additional confusion, delay and cost.

“…*making sure all your paperwork is in place and making sure you’ve got the right communication to the right regulatory body, etcetera. There’s an awful lot for you to do…”* (semi-structured interviewee 2)

One interviewee talked about the lack of regulation being a barrier for some product categories, such as digital innovations.

*“…we believe that it would be fairly straightforward to go to market with this amazing simple, inexpensive technology and we would be working from that point forward round the clock trying to keep up with the demand that the pandemic was creating. The reality is the marketplace was completely unregulated…”* (semi-structured interviewee 5)

**Fig 3:**
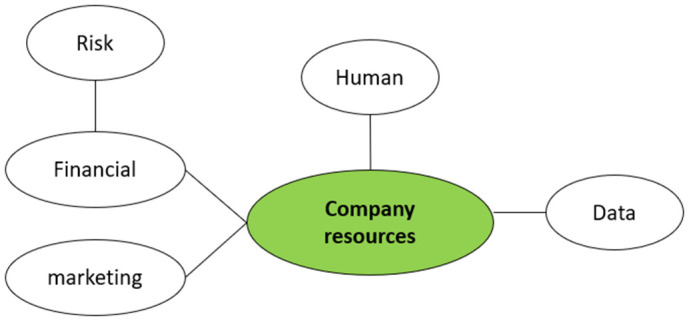
Visual representation of the 'company resources' analytical categories.

Financial Risk Limits NHS Market Access SMEs view the time and high human, financial and marketing costs to opening up the NHS market as significant business risks, as this common quote across SMEs illustrates (**Fig 3**).

*“We were very reluctant, as a small business, to embark on something which could, you know, have the very real capability of sinking our business…We have a problem that we don’t have an adequate solution for and really the call to SME’s or anybody innovative is to jog alongside us for two to five years at your own risk in order to possibly come up with something we might buy from you”.* (Semi-structured interviewee 1)

*“The expectations of the SME, e.g., social value adding, ISO compliance (…), business continuity, labor standards etc. is very hard for SME”* (Online survey 2)

Not only is the time to market a factor but also the additional data requirements for NHS adoption are both significant and highly specific, with lack of applicability to non-NHS markets. Evaluation/validation data are needed to support the product value proposition, the business case, the NHS supply chain, and to attract investors.

*“So basically, you need new data for this new thing and then of course you have worked so hard to put the data in the NHS and gathering all the packages that you have done so well and then the education sector require different data and that will mean extra work for you”.* (Semi-structured interviewee 5)

### Procurement and Supply Chain Frameworks are complex

In the UK, to facilitate procurement of goods, frameworks are used as explained by Crown Commercial Service [42] to “help public and third sector buyers to procure goods and services from a list of pre-approved suppliers, with agreed terms and conditions and legal protections”.

Having an innovative product included in the NHS supply chain framework (or any other framework) is a significant achievement and crucial for SMEs to secure their position in the NHS market and align with their long-term business plans. However, navigating the journey to join these frameworks is an intricate and challenging process for SMEs. One of the primary barriers reported by SMEs is the complexity of tenders and the substantial time and resources required to understand how the NHS procurement and supply chain operates. The intricacies of the tendering process can be overwhelming and resource-intensive, especially for companies with less than 5 employees and limited resources.

*“… a barrier has been to understand the complexities of the NHS Supply Chain process, including the level of product evidence and data…”* (Online survey 1).

NHS bureaucracy presents another challenging barrier that SMEs must overcome. Dealing with the various bureaucratic procedures and requirements can be time-consuming and frustrating for SMEs, diverting attention and resources away from core business activities.

*“There is a safety net to deal with the same suppliers in the same way and allow them to bring the innovations to market…What it means is people like us, that little, tiny minnows, specks of dust in the whole scheme of things. We have no voice”* (Semi-structured interviewee 5)

Gathering relevant evidence and data to meet the stringent requirements of the framework contracts is difficult for SMEs. The need to provide robust evidence to demonstrate the efficacy and safety of their products can be a daunting and expensive undertaking, especially for companies with limited research capabilities.

Additionally, the length of time it takes for new framework contracts to open and the limited flexibility within these contracts have been cited as significant obstacles by interviewed SMEs. The lengthy waiting period of up to three years before a new framework contract opens can hinder SMEs’ access to the market and limit their growth opportunities. This delay restricts their ability to remain flexible and agile.

**Fig 4:**
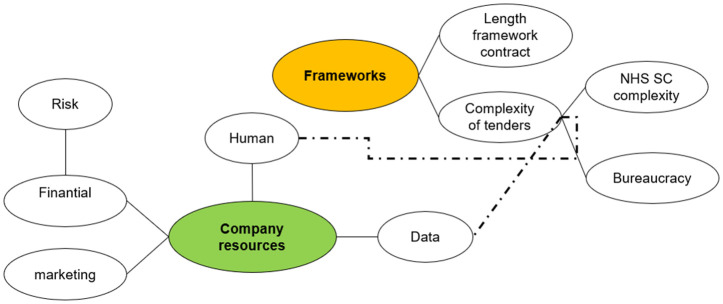
Visual representation of the connection between “company resources” and “frameworks” analytical frameworks.

Despite these challenges, SMEs remain determined to break through the barriers and make their mark in the NHS Supply Chain framework. However, as one SME mentioned, having a product listed in the NHS supply chain does not necessarily guarantee procurement. This company reported that while their product is listed, they have not been approached or received any orders due to a lack of resources for marketing and limited access to key stakeholders (**Fig 4**).

**Fig 5:**
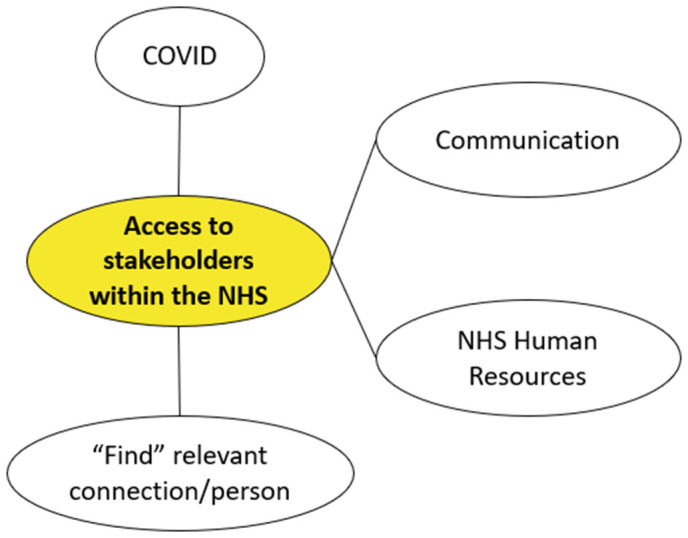
Visual representation of the “Barriers to stakeholder access within the NHS” analytical categories.

Access to NHS decision-makers lacks transparency participants in both the online survey and the interviews emphasised the extreme difficulties they faced in finding and accessing relevant NHS stakeholders for the adoption and/or procurement of a product (**Fig 5**). This includes identifying clinicians or allied health professionals who might champion the product through advocacy and/or support the SME in developing a business case, value proposition, or drafting a case study for presentation to senior management. Additionally, engaging with the procurement teams within NHS Trusts was noted as a significant challenge. Participants reported being aware the time available from clinicians to champion a product or to spend on research and innovation is limited. Access to procurement teams was, however, highlighted as being even more difficult than access to clinicians. Furthermore, some SMEs were not even aware of the roles that the different teams play within an NHS Trust.

*“Consultants who are working* [are] *so busy they don’t have time, so you can’t even have the conversation with them to explain that we’ve got the solution to their problem”.* (semi-structured interviewee 5)

*“Identifying individuals with relevant procurement responsibility and have access to them has been a barrier”* (online survey 11)

Communication channels and NHS staff availability have been affected by the COVID-19 pandemic. This, along with the multitude of available communication platforms, has posed a challenge for SMEs in arranging face-to-face meetings, which are crucial for effectively marketing their products. Moreover, NHS staff turnover has impacted on the adoption of innovative products, adding to the difficulties SMEs face.

*“…We need contact with a person in this role over a multi-year tenure, but every two years they leave, and they go somewhere else in a completely different organisation and whilst it’s still NHS, it’s completely unrelated and they can’t help anymore.”* (semi-structured interviewee 1)

NHS Decision-Making is Multifaceted The data shows four main types of stakeholders involved in NHS decision-making processes for adoption of innovative health care products – procurement and management staff, innovation teams, and clinicians. The lack of a clear hierarchy or internal pathway between them led to a lack of clarity on where to start and delays in decision-making as illustrated by these two quotes:

*“[There is] no real individual point of ownership. Collective ownership [between teams] makes decisions very difficult. Three months between board meetings means sometimes 6 months to get an answer.* (Online survey 7)

*“…there is a kind of a gap between what is the vision of the clinician champion and the adoption by the trust...”* (Semi-structure interview 4)

Nor do the various clinical, procurement and innovation teams collaborate in a comparable way between NHS trusts. If an SME would like their product to be used in different NHS Trusts for example, it will have to go through a different adoption/procurement pathway for each Trust, resulting in duplication of effort and redundancy.

*“…actually, went to the board level twice within that trust. I was quite surprised when the answer came back… as a no. And that didn’t actually happen. What then subsequently happened? We had a parallel introduction to another trust”.* (Semi-structured interviewee 1)

This experience underscores the fragmented and often unpredictable nature of the procurement process across different NHS Trusts.

## Discussion

The innovation pathway for health products is complex and multifaceted, particularly within the NHS landscape. This study, focused on the Liverpool City Region (LCR), identified common barriers faced by SMEs when trying to adopt new infection prevention and control (IPC) products into NHS settings. The findings from LCR align with broader national studies, indicating that SMEs across the UK encounter similar obstacles. These include high market-entry costs, fragmented procurement processes, and limited access to decision-makers within NHS Trusts.

These findings align with recent literature in implementation science and health innovation systems, which emphasise the influence of systemic complexity, stakeholder fragmentation, and organisational readiness on adoption outcomes [49–52]. The experiences described by SMEs in this study reflect these broader patterns, particularly the challenges of navigating fragmented NHS decision-making and securing internal champions. As McGuier et al. [49] highlight, effective teamwork is essential for successful implementation, while van de Water et al. [50] and Caci et al. [52] demonstrate how organisational readiness plays a critical role in determining whether innovations take root. Additionally, Whelan et al. [51] show that combining systems thinking with implementation science frameworks supports more effective strategy development for complex health system interventions. Our findings reinforce these insights by illustrating how structural barriers and contextual complexity within the NHS constrain SME-led innovation adoption.Similar challenges have been noted by other studies. For instance, one study emphasises that SMEs face significant hurdles in navigating regulatory pathways and addressing market-entry costs [27,28]. Additionally, another study highlights that innovations are often judged based on minimal cost, and decision-making processes within the NHS are often unclear to suppliers, further complicating access for SMEs [34]. These barriers not only limit innovation adoption but also discourage SMEs from investing in crucial health innovations.

Fragmentation within the NHS, as reflected in this study, has been highlighted in other research as a significant obstacle to innovation [3]. Different NHS Trusts have their own unique procurement processes, which necessitate SMEs to go through multiple pathways for the same product, resulting in inefficiencies and increased costs. SMEs also struggle with the complexity of the NHS as an organisation, which makes it difficult for them to access the right stakeholders who can advocate for their products within Trusts. Without strong internal networks or clinical champions, smaller enterprises find it difficult to break into the NHS market.

Moreover, the COVID-19 pandemic has exacerbated these challenges. The resulting shift in priorities, alongside resource constraints, has made it even more difficult for SMEs to engage with NHS stakeholders. This has a further complicated an already challenging market-entry landscape for SMEs seeking to offer innovative products aimed at addressing public health concerns such as antimicrobial resistance (AMR) and healthcare-associated infections (HAIs).

iiCON responded to these findings by creating a dedicated Knowledge Broker role to support SMEs in navigating the NHS innovation pathway and identifying appropriate decision-makers. Additionally, a Clinical Innovation Day was introduced to provide SMEs with a platform to showcase their technologies and connect with relevant clinical and procurement stakeholders. While these specific initiatives were not directly proposed by participants, they were developed in response to key themes identified through the study—particularly the challenges SMEs described around stakeholder access, fragmented communication, and lack of coordinated entry points into the NHS. These actions illustrate how intermediary organisations can translate research findings into targeted, practical support mechanisms.

These actions align with the national strategies outlined in “Accelerating Transformation: How to develop effective NHS-industry partnerships,” which emphasise the importance of fostering robust collaborations between the NHS and industry [53]. Moreover, engaging policymakers to address regulatory and procurement barriers, as discussed in this paper, is essential for reform.

Time constraints within the project did not allow for a comprehensive evaluation of the NHS perspective on procurement and innovation barriers. Furthermore, the relatively low response rate to the online survey may limit the representativeness of the findings. Although the data were triangulated with interview results, this limitation should be considered when interpreting the generalisability of the survey responses.

iiCON supported this study and provided access to a relevant network of SMEs within the Liverpool City Region. While this facilitated participant recruitment, the potential for perceived bias is acknowledged. To minimise this risk, the primary researcher had no prior relationship with interviewees, and a standardised approach to data collection and analysis was used. These steps were taken to ensure independence and rigour throughout the research process.

## Conclusion

SMEs play a vital role in driving innovation within the NHS, particularly in the area of infection prevention and control (IPC). However, this study highlights that the path to market for these innovative solutions is fraught with significant challenges, particularly around regulatory complexity, high market-entry costs, and fragmented NHS procurement processes. Despite these hurdles, SMEs remain motivated by the dual incentives of enhancing NHS services and achieving sustainable growth.

To capitalise on this potential, it is critical that SMEs receive targeted support to navigate the complex NHS procurement landscape. Initiatives like those implemented by iiCON, such as the introduction of a Regional Knowledge Broker and Clinical Innovation Day, provide promising frameworks to address these challenges by fostering collaboration, streamlining access to decision-makers, and improving transparency within the NHS.

Ultimately, overcoming these barriers is essential for fostering business sustainability within the healthcare sector and ensuring the NHS can adopt transformative products that enhance patient care.

The lessons learned from this study, while specific to the Liverpool City Region, resonate with broader national challenges faced by SMEs across the UK. As such, continued efforts to align SME innovation with NHS needs will be crucial for addressing both healthcare-associated infections and the growing threat of AMR.

## Supporting information

S1 File Study themes and analytical categories. (DOCX)

S2 File SMEs health survey – Semi-structure interview questions. (DOCX)

S3 File SMEs online survey. (PDF)

## Abbreviations

HAIs: Healthcare-Associated Infections
SMEs: Small and Medium Enterprises
IPC: Infection Prevention and Control
NHS: National Health Service
AMR: Antimicrobial Resistance
LCR: Liverpool City Region
HCPs: Health Care Professionals
MHRA: Medicines and Healthcare products Regulatory Agency
HLS: Health and Life Sciences
iiCON: Infection Innovation Consortium
NIHR: National Institute for Health Research
ISO: International Organization for Standardization
NVivo: Qualitative Data Analysis Software
GP: General Practitioner
COVID-19: Coronavirus Disease 2019
PPP: PowerPoint Presentation
AMPh: Antimicrobial Priorities
CIP: Coalition of Interested Parties
UKRI: UK Research and Innovation
AI: Artificial Intelligence
HLS: Health and Life Sciences
