# Correction: Overcoming barriers to NHS adoption of innovative IPC products: A qualitative study of SMEs in the Liverpool city region

**DOI:** 10.1371/journal.pone.0338734

**Published:** 2025-12-09

**Authors:** Rocío Villacorta Linaza, Janet Hemingway, Adam P. Roberts, Becky Jones-Philips, Miriam Taegtmeyer, Richard L. Wright, Daire Cantillon, Maria Moore, Russell Dacombe, Ezekiel Boro, Aaron Argomandkhah, Carolina Velasco, Nicholas Feasey

There are errors in the Author Contributions. The correct contributions are:

**Conceptualization:** Rocío Villacorta Linaza, Janet Hemingway, Adam P. Roberts, Nicholas Feasey.

**Data curation:** Rocío Villacorta Linaza.

**Formal analysis:** Rocío Villacorta Linaza, Adam P. Roberts.

**Investigation:** Rocío Villacorta Linaza, Daire Cantillon.

**Methodology:** Rocío Villacorta Linaza, Daire Cantillon.

**Project administration:** Rocío Villacorta Linaza, Daire Cantillon

**Writing – original draft:** Rocío Villacorta Linaza.

**Writing – review & editing:** Janet Hemingway, Adam P. Roberts, Becky Jones-Philips, Miriam Taegtmeyer, Richard L. Wright, Daire Cantillon, Maria Moore, Russell Dacombe, Ezekiel Boro, Aaron Argomandkhah, Carolina Velasco, Nicholas Feasey.
