# Supplementary material for: Overcoming barriers to NHS adoption of innovative IPC products: A qualitative study of SMEs in the Liverpool city region
Source: PLoS One. 2025 Sep 16;20(9):e0331688. doi: 10.1371/journal.pone.0331688 (PMC12440186; doi:10.1371/journal.pone.0331688)
Supplement: S1 File — (DOCX) [file pone.0331688.s001.docx]

**Supporting information**

Study themes and analytical categories

| **Themes** | **Analytical categories** |
| --- | --- |
| **Cost** | Ownership of Intelectual Property |
|  | Regulation for innovation products |
|  | Competing with established brands |
|  | Funding available for SMEs with Digital applications |
| **Company resources** | Human |
|  | Economical |
|  | Marketing |
|  | Risk |
|  | Access to relevant data |
| **Procurement and supply chain frameworks** | Length of framework contract |
|  | Complexity of tenders |
| **Access to NHS**  **stakeholders** | Communication channels between SMEs and NHS stakeholders |
|  | COVID-19 |
|  | Find “relevant” person |
|  | NHS HR |
| **Decision-making process** | Procurement |
|  | Clinicians |
|  | Innovation |
|  | Management |
