# Supplementary material for: Overcoming barriers to NHS adoption of innovative IPC products: A qualitative study of SMEs in the Liverpool city region
Source: PLoS One. 2025 Sep 16;20(9):e0331688. doi: 10.1371/journal.pone.0331688 (PMC12440186; doi:10.1371/journal.pone.0331688)
Supplement: S2 File — (DOCX) [file pone.0331688.s002.docx]

Bloomsbury SET

*SMEs health survey*

Semi-structure interview questions

1. Could you please describe the work your company does?
2. Could you please explain the barriers you have/are facing while trying to adopt your product to the NHS?
3. Could you explain your experience working with different trust and if the barriers/challenges have been the same for each trust?
4. If you have more than 2 products adopted by the NHS, did you face same barriers/challenges for each product?
5. Any barrier for being a infection disease product?
