# Supplementary material for: Overcoming barriers to NHS adoption of innovative IPC products: A qualitative study of SMEs in the Liverpool city region
Source: PLoS One. 2025 Sep 16;20(9):e0331688. doi: 10.1371/journal.pone.0331688 (PMC12440186; doi:10.1371/journal.pone.0331688)
Supplement: S3 File — (PDF) [file pone.0331688.s003.pdf]

SMEs questionnaire

Thank you for taking approximately 20 minutes to complete this survey.

Navigating the NHS Innovation Pathway towards NHS Adoption for any company (SME) or organisation is a complex and often daunting task. The purpose of this survey is to understand your individual pathway journey and to identify specific barriers or obstacles SMEs have, or currently are, facing in getting products through to procurement in the health care sector. This includes the NHS (including dental practices) as well as private healthcare providers.

LSTM aims to use these data to formulate strategies to assist SMEs in overcoming these bottlenecks that prevent products getting through to market.

All information will be treated as strictly confidential, and no responses will be associated with a specific individual or company. Results will be made available to SMEs who participate in the form of a published report.

Please start by answering the question below.

1

What is the legal status of your organisation?

- ○ Sole proprietorship
- ○ Private limited company, limited by shares (LTD.)
- ○ Public Ltd Company (PLC)
- ○ Partnership
- ○ Limited liability partnership
- ○ Private company limited by guarantee
- ○ Community Interest Company (CIC, limited by guarantee or shares)
- ○ Friendly Society
- ○ A Co-operative
- ○ Industrial and Provident Society
- ○ Private Unlimited Company
- ○ Foreign Company
- ○ A trust
- ○ An unincorporated association
- ○ Other

2

What year was your company established?

3

Is your company based in the Merseyside?

- ◯ Yes
- ◯ No

4

How many full-time equivalent employees does your company employ today?

- ◯ 1-5
- ◯ 6-10
- ◯ 11-50
- ◯ 51-100
- ◯ More than 100
- ◯ we are a virtual company with zero full-time equivalent employees
- ◯ I don't know

5

How many products are you developing to be adopted by the NHS?

- 1
- 2
- 3
- 4
- More than 5
- None

6

Are any of these products an innovative infection control or prevention product?

- Yes
- No

7

Please provide a brief description of your product(s) (e.g. biocide, surface cleaner, dressings etc)

8

What market are you targeting for your product? Select all that apply.

- ☐ NHS trusts
- ☐ Community services
- ☐ Private health care
- ☐ Specialist trusts
- ☐ Primary health care (GP surgeries)
- ☐ Dentists
- ☐ Wholesalers
- ☐ Cleaning companies
- ☐ Residential care providers
- ☐ Other

9

At which point of the development stage is your product?

- ○ Compound
- ○ Testing
- ○ Regulation
- ○ Subscription
- ○ Market
- ○ Other

10

If your product has been or is in the testing phase, what challenges have you faced or currently are facing?

11

Have you had any support from any of the following local, national or alternatives innovation initiatives?

- [ ] Local Enterprise Partnership/Local Council/Growth Platform/Growth Hub
- [ ] Innovate UK
- [ ] NHS Accelerated Access Collaborative (AAC)
- [ ] NHS Innovation Accelerator
- [ ] Academic Health Sciences Centres
- [ ] Academic Health Science Networks
- [ ] Regional Medicines Optimisation Committees
- [ ] NHS Digital
- [ ] NHSX
- [ ] Medilink UK

12

Have you have any financial support from any of the following organisations?

- ☐ NIHR Funding Schemes
- ☐ NHS Innovation Accelerator
- ☐ Innovate UK
- ☐ Angel Investors
- ☐ Venture Capital
- ☐ Start-up Accelerators/Incubators

13

If your product requires regulatory compliance, has this been achieved?

- ○ Yes
- ○ No

14

Who have you engaged within the NHS during the product development phase?

- ☐ NHS trust - Clinicians
- ☐ NHS trust - procurement department
- ☐ NHS Supply Chain
- ☐ Primary health care (GP surgeries)
- ☐ External companies that work for NHS
- ☐ Other

15

Please describe up to three challenges you have or are facing while putting together your product value proposition

16

Which of the following marketing strategies have you employed while developing your product?

- [ ] None
- [ ] Email
- [ ] Social media
- [ ] Pay media advertising
- [ ] Content marketing
- [ ] Face to face direct talk
- [ ] Traditional marketing such as radio, newspapers, leaflets
- [ ] Other

17

What capacity does your SME possess for developing marketing strategies? This may include staffing resources to develop marketing; funding to outsource marketing requirements; staff expertise to develop marketing etc.

18

What are the routes to market you have engaged with?

- ☐ Selling direct to trusts or primary care organisations
- ☐ Selling through collaborative purchasing arrangements
- ☐ Crown commercial services
- ☐ Government tenders and contracts
- ☐ NHS Supply Chain (framework towers)
- ☐ Other

19

Which of the following marketing routes within the NHS are you aware of:

- ☐ eDirect
- ☐ Blue Diamond
- ☐ Stocked products
- ☐ None of the above

20

Please refer to the diagram below, which details an overview of the procurement process.

For the NHS, one of the first step to get into this procurement process is to have your product included in the NHS supply chain database/catalogue. Please describe the main barrier(s) you have faced in getting access to this catalogue.

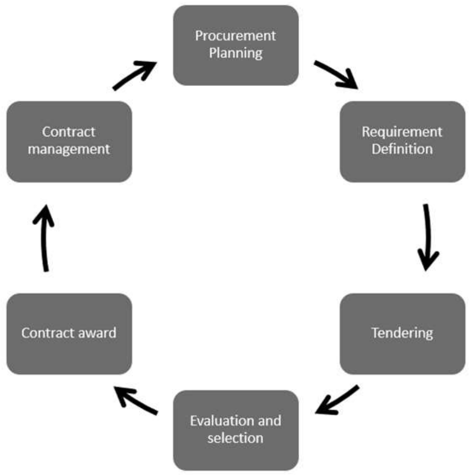

21

Describe the *three biggest* challenges you have faced in getting products to market within the NHS. This may include products you have succeeded in getting to market or those that have been unsuccessful.

22

Have you been able to sell your product(s) to any of the following wholesalers that supplies to primary care (GP surgeries, health centres, pharmacies, dentists) or/and companies that provide services to the NHS (e.g. cleaning companies)?

- ○ AHH
- ○ Alliance
- ○ Phoenix
- ○ Sigma
- ○ Trident
- ○ OTC direct
- ○ Colorama
- ○ Other

23

How could an academic partner like iiCON contribute to accelerating products through to procurement in the health care sector?

24

Free text comment on any other barrier or aspect that you have faced before or during the procurement process discussed above:

This content is neither created nor endorsed by Microsoft. The data you submit will be sent to the form owner.

Microsoft Forms
